# Arbaclofen extended-release tablets for spasticity in multiple sclerosis: open-label extension study

**DOI:** 10.1093/braincomms/fcad026

**Published:** 2023-02-07

**Authors:** Darin T Okuda, Daniel Kantor, Mark Jaros, Tina deVries, Samuel Hunter

**Affiliations:** The University of Texas Southwestern Medical Center, Dallas, TX 75390-8806, USA; Florida Atlantic University, Boca Raton, FL 33431, USA; Nova Southeastern University, Fort Lauderdale, FL 33314, USA; Summit Analytical, Denver, CO 80238, USA; RVL Pharmaceuticals, Inc., Bridgewater, NJ 08807, USA; Advanced Neuroscience Institute, Franklin, TN 37064, USA

**Keywords:** spasticity, multiple sclerosis, arbaclofen, extended-release, baclofen

## Abstract

Baclofen, a racemic γ-aminobutyric acid B receptor agonist, is commonly used for the management of multiple sclerosis–related spasticity but is associated with frequent dosing and poor tolerability. Arbaclofen, the active R-enantiomer of baclofen, exhibits 100- to 1000-fold greater specificity for the γ-aminobutyric acid B receptor compared with the S-enantiomer and ∼5-fold greater potency compared with racemic baclofen. Arbaclofen extended-release tablets allow a dosing interval of 12 h and have shown a favourable safety and efficacy profile in early clinical development. A 12-week, randomized, placebo-controlled Phase 3 trial in adults with multiple sclerosis–related spasticity demonstrated that arbaclofen extended-release 40 mg/day significantly reduced spasticity symptoms compared with placebo and was safe and well tolerated. The current study is an open-label extension of the Phase 3 trial designed to evaluate the long-term safety and efficacy of arbaclofen extended-release. In a 52-week, open-label, multicentre study, adults with a Total Numeric-transformed Modified Ashworth Scale score ≥2 in the most affected limb received oral arbaclofen extended-release titrated over 9 days up to 80 mg/day based on tolerability. The primary objective was assessment of arbaclofen extended-release safety and tolerability. Secondary objectives included an assessment of efficacy using the Total Numeric-transformed Modified Ashworth Scale-most affected limb, the Patient Global Impression of Change and Expanded Disability Status Scale. Of 323 patients enrolled, 218 (67.5%) completed 1 year of treatment. Most patients (74.0%) achieved an arbaclofen extended-release maintenance dose of 80 mg/day. At least one treatment-emergent adverse event was reported by 278 patients (86.1%). The most common adverse events were [*n* patients (%)]: urinary tract disorder [112 (34.7)], muscle weakness [77 (23.8)], asthenia [61 (18.9)], nausea [70 (21.7)], dizziness [52 (16.1)], somnolence [41 (12.7)], vomiting [29 (9.0)], headache [24 (7.4)] and gait disturbance [20 (6.2)]. Most adverse events were of mild–moderate severity. Twenty-eight serious adverse events were reported. One death occurred during the study, a myocardial infarction that was considered by investigators as unlikely to be related to treatment. Overall, 14.9% of patients discontinued due to adverse events, primarily muscle weakness, multiple sclerosis relapse, asthenia and nausea. Evidence of improvement in multiple sclerosis–related spasticity was observed across arbaclofen extended-release dosages. Arbaclofen extended-release treatment (up to 80 mg/day) was well tolerated and reduced symptoms of spasticity in adult patients with multiple sclerosis for 1 year.

*Clinical Trial Identifier:* ClinicalTrials.gov, NCT03319732

## Teaser

This 52-week, open-label, multicentre study reported by Okuda *et al.* assessed safety and efficacy of up to 80 mg/day arbaclofen extended-release in adults with multiple sclerosis–related spasticity. Treatment-emergent adverse events were reported by 86.1% of patients. Most were of mild–moderate severity and commonly included muscle weakness and urinary tract infection.

## Graphical Abstract

**Graphical Abstract:**
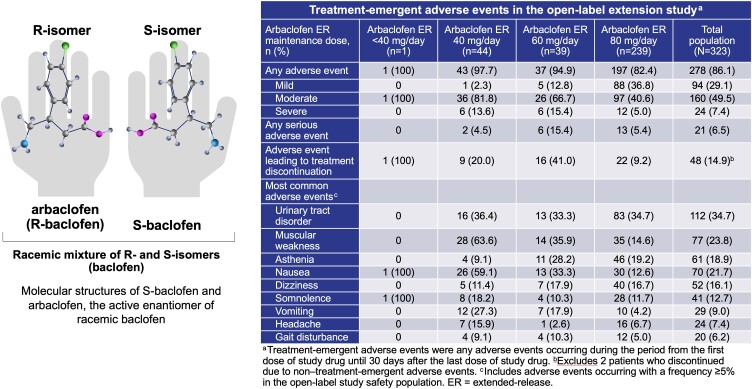

## Introduction

Multiple sclerosis is a chronic, debilitating, inflammatory disease of the CNS affecting approximately 900 000 patients in the USA.^1,2^ As many as 80% of patients with multiple sclerosis experience spasticity, an involuntary contraction or shortening of muscle tissues caused by impaired transmission of inhibitory impulses via nerve fibres in the CNS.^3–8^ Findings on clinical examination include resistance to passive movement, velocity-dependent increases in tonic stretch reflexes and decreased range of motion.^4,9^ Spasticity is associated with pain, fatigue, gait disturbance, impaired sleep and bladder dysfunction.^3,10^ One-third of patients with multiple sclerosis experience moderate, severe or total limitation in physical ability as a result of spasticity.^5^

The most commonly prescribed treatment for the management of multiple sclerosis–related spasticity is baclofen, a racemic γ-aminobutyric acid B (GABA_B_) receptor agonist that inhibits excitatory signal transmission via supraspinal and spinal cord synapses.^4,11^ Clinical studies evaluating baclofen in patients with multiple sclerosis have shown improvements in various measures of spasticity compared with placebo.^12–14^ However, the therapeutic potential of baclofen is limited by poor transport across the blood–brain barrier and poor tolerability owing to adverse events, such as sedation, drowsiness and worsening fatigue.^5,6,11,15^ Additionally, baclofen is administered in divided doses up to four times daily, increasing the potential for suboptimal compliance.

Evidence from *in vitro* and animal studies suggest that the therapeutic effect of racemic baclofen is primarily attributable to the active R-enantiomer, arbaclofen.^16–19^ Arbaclofen exhibits 100- to 1000-fold greater specificity for the GABA_B_ receptor compared with the S-enantiomer and approximately 5-fold greater potency compared with racemic baclofen.^18,20–23^ Arbaclofen extended-release (ER) employs osmotic pump technology to provide controlled release and sustained concentrations of drug, allowing a dosing interval of 12 h. Arbaclofen ER doses ranging from 10 to 80 mg/day for up to 20 days were generally well tolerated in Phase 1 studies of healthy volunteers (data on file, RVL Pharmaceuticals, 2017).

The Phase 3 clinical trial programme for arbaclofen ER tablets was comprised of two randomized controlled studies and two open-label studies in patients with spasticity related to multiple sclerosis. Here, we report the results of a multinational, open-label study of arbaclofen ER 40 and 80 mg/day in patients with multiple sclerosis–related spasticity. The study was conducted to determine safety, tolerability, efficacy and patient-reported outcome data associated with long-term arbaclofen ER treatment from participants involved in the arbaclofen ER clinical development programme.

## Materials and methods

### Study design and patient population

The open-label, long-term study was conducted at 67 sites in 10 countries (the USA, Russia, Belarus, Serbia, Bosnia and Herzegovina, Croatia, Bulgaria, Moldova, Poland and Ukraine). Eligible patients received a maintenance dose of arbaclofen ER orally twice daily for 52 weeks. Doses were increased over 9 days to the highest tolerated dose with a maximal dose of 80 mg/day as follows: treatment started with 40 mg/day (2 × 20 mg) for 3 days, followed by 60 mg/day (1 × 20 mg and 1 × 40 mg) for 3 days and then 80 mg/day (2 × 40 mg). During the 52-week maintenance period, dose reduction and subsequent re-titration were permitted on three separate occasions for patients with tolerability concerns. At the end of the study, there was a 2-week taper period. Patients completed study visits at baseline (Day 92 of treatment for patients who rolled over from the randomized controlled study; NCT03290131) and at Weeks 2, 6, 14, 28, 42, 54 and 60.

The open-label study population included patients who completed the randomized, double-blind, placebo-controlled, 12-week trial (NCT03290131) and *de novo* patients. A complete list of inclusion and exclusion criteria is available in the online supplement (Supplementary Methods, online supplement). Eligible patients were adults (age, 18–65 years) with an established diagnosis of multiple sclerosis according to McDonald criteria,^24^ a documented history of spasticity for at least 6 months prior to screening, a Total Numeric-transformed Modified Ashworth Scale (TNmAS) score ≥2 in the most affected limb (MAL) and an Expanded Disability Status Scale (EDSS) score ≥3. Patients taking medications indicated for the treatment of spasticity were required to complete a washout period of up to 21 days prior to randomization in the 12-week controlled study (rollover subjects) or enrollment in the open-label (*de novo* subjects). Exclusion criteria included use of high-dose oral or intravenous methylprednisolone, or equivalent, within 3 months of baseline measures as well as the use of concomitant medications that might potentially interfere with the arbaclofen ER or outcome variables. Concomitant treatment with disease-modifying medications was permitted provided there was no change in dose for at least 3 months prior to screening and the patient was willing to maintain the dose for the duration of the study.

This study was conducted according to the principles set forth in the Declaration of Helsinki. The study protocol was approved by the institutional review board or ethics committee at each participating study site. All patients provided written informed consent prior to enrollment.

### Study outcomes

The primary objective of this study was to evaluate the long-term safety and tolerability of arbaclofen ER. Safety assessments included adverse events, vital signs, clinical laboratory tests, 12-lead electrocardiograms, the Urinary Symptom Profile Questionnaire^25^ and the Columbia-Suicide Severity Rating Scale.^26^

Secondary objectives were the assessment of efficacy at 1 year using the TNmAS, Patient Global Impression of Change (PGIC) and EDSS. The TNmAS and EDSS scores were measured at baseline and at Weeks 28 and 60; PGIC was measured at Week 60. The TNmAS is a validated 6-point rating scale evaluating the degree of abnormality in muscle tone or the resistance to passive movements; higher scores indicate more severe spasticity.^27,28^ The TNmAS was calculated by the evaluator measuring the modified Ashworth score at each of the following joints bilaterally: shoulder, elbow, wrist, hip, knee and ankle. The sum of the scores for each limb’s three joints was used to determine the MAL. A total limbs score for the entire exam of 12 joints was also calculated. Although the study was open-label, assessments were performed by a trained evaluator who was blinded to treatment assignment and all clinical, laboratory and safety assessments. The PGIC is a 7-point scale that measures the change in activity limitations, symptoms and quality of life; higher scores indicate greater overall improvement. The EDSS quantifies multiple sclerosis–related disability on a scale ranging from 0 (normal neurological exam) to 10 (death due to multiple sclerosis).^29^

### Statistical methods

Outcomes were reported as the mean change from baseline at each assessment interval for TNmAS and EDSS scores and as the mean score at the final study visit for the PGIC score.

All safety and efficacy analyses were conducted in the safety population, which included any patient who received at least one dose of the study drug and had at least one post-dose visit. Analyses were performed using SAS® software version 9.4 (SAS Institute, Cary, NC). Adverse events were coded according to preferred terms in the *Medical Dictionary for Regulatory Activities*, version 20.1. Safety outcomes were reported as events occurring between the first dose of study treatment and 30 days after the last dose of study treatment. A data safety monitoring board comprising four experienced multiple sclerosis clinicians prospectively reviewed comprehensive, unblinded safety datasets, including, but not limited to, clinical laboratory data and adverse events, throughout the duration of the study.

### Data availability

The data that support the findings of this study are not publicly available due to confidentiality restrictions.

## Results

**Figure 1:**
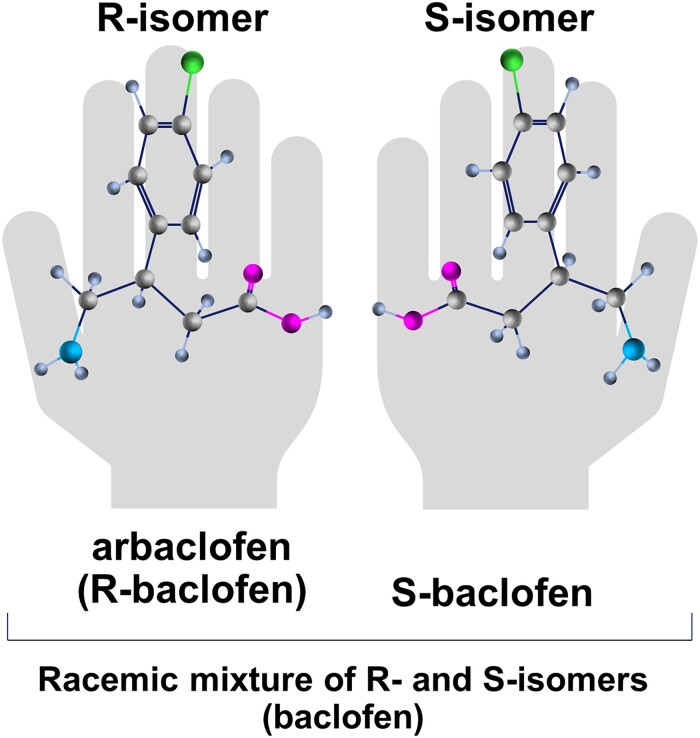
Molecular structures of the R-isomer (arbaclofen), the active enantiomer of racemic baclofen, and the S-isomer.

A total of 323 patients enrolled in the open-label study (March 2018 to January 2020), including 317 (98.1%) patients who completed the randomized controlled study (NCT03290131) and 6 (1.9%) *de novo* patients (Table 1 and Fig. 1, online supplement). A majority of patients (74.0%) achieved an arbaclofen ER maintenance dose of 80 mg/day during the 9-day titration period; among the remaining patients, 44 (13.6%) received a maintenance dose of 40 mg/day, and 39 (12.1%) received 60 mg/day. One (0.3%) patient received <40 mg/day and subsequently discontinued. A total of 218 (67.5%) patients completed the 12-month open-label study, including 172 of 239 (72.0%) patients who received a maintenance dose of 80 mg/day. Of the 181 patients who received arbaclofen ER in the randomized controlled study (i.e. treatment-experienced), 142 (78.5%) completed the open-label study compared with 76 (53.5%) of 142 patients who were *de novo* or received placebo in the randomized controlled study (i.e. treatment-naïve). The majority of discontinuations [105/323 (32.5%)] were due to patient request [50 (15.4%)], treatment-emergent adverse events [48 (14.9%)] or relapse of multiple sclerosis symptoms [10 (3.1%)].

**Table 1:** Demographics and baseline characteristics

Characteristic	Total population (*N* = 323)
Age, mean (SD), years	47.2 (9.31)
Sex, *n* (%)	
Female	190 (58.8)
Male	133 (41.2)
Race, *n* (%)	
Asian	0
Black or African American	6 (1.9)
Caucasian	312 (96.6)
Mixed	1 (0.3)
Not collected	4 (1.2)
Height, mean (SD), cm	169.6 (8.94)
Weight, mean (SD), kg	71.7 (15.70)
Body mass index, mean (SD), kg/m^2^	24.8 (4.80)
MS subtype, *n* (%)	
Relapsing–remitting	205 (64.7)^a^
Primary progressive	17 (5.4)^a^
Secondary progressive	95 (30.0)^a^
TNmAS-MAL score, mean (SD)	6.3 (3.25)
Prior therapy, *n* (%)	
Baclofen	11 (3.4)
Diazepam	0
Gabapentin	1 (0.3)
Tizanidine	0

a MS subtype was not recorded for the six *de novo* subjects. MS, multiple sclerosis; SD, standard deviation; TNmAS-MAL, Total Numeric-transformed Modified Ashworth Scale-most affected limb.

### Safety outcomes

A total of 278 (86.1%) patients experienced at least one adverse event during the open-label study, most of which were of mild-to-moderate severity (Table 2). The most common adverse event was urinary tract disorder, occurring in 112 (34.7%) patients. Other adverse events occurring with a frequency >10% in the overall population were muscular weakness [77 (23.8%) patients], nausea [70 (21.7%) patients], asthenia [61 (18.9%) patients], dizziness [52 (16.1%) patients] and somnolence [41 (12.7%) patients]. The incidence of moderate and severe adverse events was lower among patients receiving arbaclofen ER 80 mg/day compared with the lower doses. Three of these patients discontinued due to non­–treatment-emergent adverse events. Fewer patients who received arbaclofen ER 80 mg/day discontinued treatment (28.0%) compared with those who received lower doses (40.9 and 48.7% in the 40 and 60 mg/day groups, respectively; Supplementary Table 1, online supplement). Adverse events led to discontinuation in 14 (7.7%) treatment-experienced patients and 36 (25.4%) treatment-naïve patients (Supplementary Table 2, online supplement). There were no clinically significant changes in mean laboratory values or vital signs. Electrocardiogram findings were unremarkable, with the exception of one patient who experienced a clinically significant electrocardiogram abnormality consistent with mild bradycardia at the Week 28 and Week 60 study visits.

**Table 2:** Treatment-emergent adverse events in the open-label extension study ^a^

Arbaclofen ER maintenance dose, *n* (%)	Arbaclofen ER	Arbaclofen ER	Arbaclofen ER	Arbaclofen ER	Total population (*N* = 323)
	<40 mg/day (*n* = 1)	40 mg/day (*n* = 44)	60 mg/day (*n* = 39)	80 mg/day (*n* = 239)	
Any adverse event	1 (100)	43 (97.7)	37 (94.9)	197 (82.4)	278 (86.1)
Mild	0	1 (2.3)	5 (12.8)	88 (36.8)	94 (29.1)
Moderate	1 (100)	36 (81.8)	26 (66.7)	97 (40.6)	160 (49.5)
Severe	0	6 (13.6)	6 (15.4)	12 (5.0)	24 (7.4)
Any serious adverse event	0	2 (4.5)	6 (15.4)	13 (5.4)	21 (6.5)
Adverse event leading to treatment discontinuation	1 (100)	9 (20.0)	16 (41.0)	22 (9.2)	48 (14.9)^b^
Most common adverse events ^c^					
Urinary tract disorder	0	16 (36.4)	13 (33.3)	83 (34.7)	112 (34.7)
Muscular weakness	0	28 (63.6)	14 (35.9)	35 (14.6)	77 (23.8)
Asthenia	0	4 (9.1)	11 (28.2)	46 (19.2)	61 (18.9)
Nausea	1 (100)	26 (59.1)	13 (33.3)	30 (12.6)	70 (21.7)
Dizziness	0	5 (11.4)	7 (17.9)	40 (16.7)	52 (16.1)
Somnolence	1 (100)	8 (18.2)	4 (10.3)	28 (11.7)	41 (12.7)
Vomiting	0	12 (27.3)	7 (17.9)	10 (4.2)	29 (9.0)
Headache	0	7 (15.9)	1 (2.6)	16 (6.7)	24 (7.4)
Gait disturbance	0	4 (9.1)	4 (10.3)	12 (5.0)	20 (6.2)

a Treatment-emergent adverse events were any adverse events occurring during the period from the first dose of study drug until 30 days after the last dose of study drug. ^b^Excludes two patients who discontinued due to non–treatment-emergent adverse events. ^c^Includes adverse events occurring with a frequency ≥5% in the open-label study safety population. ER, extended-release.

A total of 28 serious adverse events occurred in 21 (6.5%) patients. The only serious adverse event that occurred in more than one patient was relapse of multiple sclerosis [7 (2.2%) patients]. All cases of multiple sclerosis relapse were assessed by the investigator as unlikely related to treatment. All serious adverse events resolved except for a fatal acute myocardial infarction that occurred in a 53-year-old male that was assessed by investigators as unlikely related to study treatment. One patient receiving arbaclofen ER 80 mg/day reported mild, fleeting suicidal ideation at Week 2 (Day 15) with no consequential sequelae. There was no evidence of worsening in urinary symptoms as measured by mean changes in parameter values from baseline to post-baseline study visit on the Urinary Symptom Profile Questionnaire (Supplementary Table 3, online supplement).

### Efficacy outcomes

At Week 28, the overall mean [standard deviation (SD)] change from open-label extension baseline in TNmAS-MAL score was −0.7 (1.73), with evidence of improvement observed across all dose groups when compared to baseline measures from the randomized controlled trial (Supplementary Table 4, online supplement). At Week 60, 4 weeks after tapering from study drug, the overall mean (SD) change from baseline was −0.1 (1.70). Among patients who completed the randomized controlled trial, the mean (SD) change in TNmAS-MAL score as measured from baseline in the randomized controlled study was −2.0 (2.16) at Week 28 and −1.5 (2.13) at Week 60, with improvements observed in all dose groups at both time points (Supplementary Table 4, online supplement). A similar pattern was observed for the TNmAS-total limbs score (Table 3; Supplementary Table 4, online supplement).

**Table 3:** Efficacy outcomes in the open-label extension study

Outcome, mean (SD)	Arbaclofen ER	Arbaclofen ER	Arbaclofen ER	Total population (*N* = 323)^a^
	40 mg/day (*n* = 44)	60 mg/day (*n* = 39)	80 mg/day (*n* = 239)	
TNmAS-MAL score				
Baseline^b^	5.6 (3.46)	5.9 (3.38)	6.5 (3.18)	6.3 (3.25)
Week 28	4.9 (3.31)	5.6 (3.44)	5.7 (3.16)	5.6 (3.22)
Week 28 change from baseline	−0.6 (1.85)	−0.4 (1.44)	−0.7 (1.75)	−0.7 (1.73)
Week 60^c^	5.1 (3.24)	5.5 (3.56)	6.3 (3.28)	6.0 (3.31)
Week 60 change from baseline	0.0 (1.80)	−0.2 (1.69)	−0.1 (1.69)	−0.1 (1.70)
TNmAS-TL score				
Baseline^b^	11.1 (9.24)	11.3 (8.30)	13.7 (7.73)	13.0 (8.06)
Week 28	9.5 (8.65)	11.0 (8.65)	12.0 (7.42)	11.5 (7.77)
Week 28 change from baseline	−1.4 (4.89)	−0.4 (2.77)	−1.4 (3.73)	−1.3 (3.81)
Week 60^c^	11.0 (10.28)	10.2 (7.65)	13.4 (8.03)	12.8 (8.36)
Week 60 change from baseline	0.3 (5.90)	0.4 (2.78)	0.3 (3.98)	0.3 (4.18)
PGIC score				
Baseline^b^	2.7 (1.53)	3.3 (1.87)	3.4 (1.87)	3.3 (1.61)
Week 60^c^	2.0 (1.34)	2.4 (1.65)	2.9 (1.63)	2.7 (1.63)
EDSS score				
Baseline^b^	4.90 (1.47)	5.21 (1.29)	4.96 (1.25)	4.98 (1.29)
Week 28	4.77 (1.46)	5.03 (1.29)	5.01 (1.27)	4.98 (1.29)
Week 28 change from baseline	0.03 (0.29)	0.15 (0.65)	0.06 (0.31)	0.06 (0.35)
Week 60^c^	4.83 (1.41)	5.47 (1.23)	4.98 (1.28)	5.01 (1.30)
Week 60 change from baseline	0.07 (0.30)	0.32 (0.65)	0.04 (0.27)	0.08 (0.35)

a Includes one patient who received <40 mg and discontinued treatment due to nausea categorized as a moderate adverse event. ^b^Baseline = Week 13 of the randomized controlled trial for rollover patients and Visit 1 for *de novo* patients. ^c^Assessment performed 4 weeks after tapering from study drug. EDSS, Expanded Disability Status Scale; ER, extended-release; PGIC, Patient Global Impression of Change; SD, standard deviation; TNmAS-MAL, Total Numeric-modified Ashworth Scale-most affected limb; TNmAS-TL, Total Numeric-modified Ashworth Scale-total limbs.

Patients’ self-reported overall status as measured by PGIC scores 4 weeks after tapering from study treatment suggested a slight reduction from baseline (Week 13 of the randomized controlled trial for rollover patients) in all treatment groups (Table 3).

Mean scores on the EDSS remained stable throughout the open-label study, demonstrating no worsening of disability over 12 months of treatment (Table 3). These results were consistent with those seen in the randomized controlled trial.

## Discussion

In this multinational, open-label study, arbaclofen ER at doses of up to 80 mg/day was generally well tolerated and considered by investigators to be safe. A majority of patients were able to titrate up to a maintenance treatment dose of 80 mg/day. Our results demonstrated a well-characterized safety profile with no evidence of new or unexpected adverse events identified during treatment for up to 1 year with 80 mg/day arbaclofen ER. Adverse events were generally mild to moderate and without significant clinical consequence, occurring at a frequency similar to previously performed studies.^30,31^ Urinary tract disorders occurred at a frequency similar to the observed frequency in the placebo group during the randomized controlled study. Reductions in TNmAS scores during the randomized controlled trial were observed during this open-label study, with a slight improvement seen at Week 28 and a reduction in the magnitude of improvement 4 weeks after tapering from study treatment. The magnitude of improvement during treatment was generally consistent with that observed in the randomized controlled study. These findings add to the previously reported information from an earlier placebo-controlled trial and open-label study of arbaclofen ER 40 mg/day.^30,31^

The management of spasticity in patients with multiple sclerosis represents a distinct challenge due to the complex and heterogenous nature of the symptoms and limitations of current therapies. Commonly prescribed treatments such as baclofen and tizanidine are associated with poor tolerability, particularly during long-term use, resulting in suboptimal dosing and limited therapeutic benefit.^5–7,11,32^ Notably, in a cross-sectional survey of more than 10 000 patients with multiple sclerosis, fewer than 50% of patients receiving treatment for spasticity expressed satisfaction with their treatment.^4^ In the context of the unmet need for a safe and efficacious therapy with proven long-term tolerability, the safety findings from the open-label study are noteworthy. Investigators in the open-label study had the discretion to temporarily taper the dose on three distinct occasions to manage issues related to tolerability, reflecting typical clinical practice and, thereby, improving the generalizability of the findings. These data also highlight the challenges associated with trials aimed as symptomatic management as a pre-defined target dose for study may not be ideal for all study patients with multiple sclerosis. In this study, the overall mean EDSS scores were unchanged, and the mean changes from baseline were small (less than 0.10) suggesting that patient disability did not worsen during the course of arbaclofen ER treatment.

Interestingly, we identified lower moderate and severe adverse events and lower discontinuation rates (28.0%) in the arbaclofen ER 80 mg/day group than the 40 mg/day (40.9%) and 60 mg/day (48.7%) groups. Muscular weakness, an outcome with an anticipated higher frequency in the 80 mg/day group, was also found to be highest in those on 40 mg/day (63.6% for 40 mg/day, 35.9% for 60 mg/day and 14.6% for 80 mg/day). In contrast to the incidence of adverse events reported in the randomized controlled trial (also presented in this issue), none of the most common adverse events increased with increasing dose in this open-label study. Similarly, in a separate multinational, open-label study which assessed arbaclofen ER at dosages of up to 40 mg/day, the most common adverse events did not generally increase with increasing dose of arbaclofen ER.^30,31^ Difference in dose response with respect to adverse events seen in randomized controlled trials and open-label studies may be due to the fact that temporary dose tapering was permitted in the open-label studies but not in the randomized controlled trials. Thus, the safety profile of the open-label trial may be like what could be expected in clinical practice. Animal studies and *in vitro* evidence suggest that the therapeutic effect of racemic baclofen is primarily attributable to the active R-enantiomer, arbaclofen.^16–19^ Arbaclofen demonstrates increased specificity and potency for the GABA_B_ receptor compared with racemic baclofen.^18,20–23^ A previous multicentre, randomized placebo- and active-controlled trial demonstrated that 40 mg/day arbaclofen ER had similar efficacy to 80 mg/day baclofen.^30^ Because the pharmacokinetics and pharmacodynamic effects of arbaclofen ER differ from racemic baclofen preparations, there is neither a fixed weight nor a molar equivalence to the R-enantiomer in a racemic preparation proportional to circulation of drug or effect. Although this is principally due to bioavailability differences of the ER preparation, other factors involving interactions between enantiomers are also plausible. Future studies will be needed to determine to what extent the long-term safety and efficacy profile of arbaclofen ER is due to increased specificity and potency for GABA_B_ receptors, decreased dosing and/or reduced off-target effects.

Final efficacy assessments in this study occurred at Week 60, 4 weeks after patients discontinued therapy. Across arbaclofen doses, improvements in TNmAS-MAL scores reported through Week 28 [mean (SD) change from baseline in the randomized controlled study = −2.0 (2.16)] were reduced at Week 60 [−1.5 (2.13)]. This demonstrates the durability of efficacy and reflects the pharmacodynamic profile of arbaclofen ER.

Our open-label data should be evaluated in the context of a few limitations. Of the 403 persons with multiple sclerosis who completed the Phase 3 randomized controlled study, 323 (80.1%) opted for participation in the open-label trial. Therefore, although the blind was maintained, our reported data may have been biased by including those who previously had a perceived positive response to treatment coupled with the selective dropout of those with a perceived lack of efficacy. In addition, the majority [218 (67.5%)] of open-label participants completed 1 year of treatment, further highlighting the challenges of the balance between therapeutic efficacy and treatment intolerance along with adverse reactions. In addition, as many of the study participants were studied from Eastern European countries, key factors such as social determinants of health and support networks may have influenced study outcomes. The treatment effects in non-white groups are also still unknown. Our data were also limited by a lack of effective instruments that quantify all relevant aspect of multiple sclerosis spasticity. However, despite this, we did identify clinically meaningful improvement in TNmAS-MAL (and TNmAS-total limbs) for all tested doses of arbaclofen ER over the duration of the study. Lastly, the titration of the arbaclofen ER dose and protocol used to address symptoms related to tolerability may not be similar to clinical practice around the world. However, slower titration schedules and more adaptive approaches to reduce the incidence of adverse reactions, while providing meaningful effect on negative multiple sclerosis symptoms, likely would have only led to better outcomes than those presented here.

In conclusion, long-term treatment with arbaclofen ER at doses up to 80 mg/day was generally well tolerated and resulted in sustained improvements in spasticity. Considering the well-characterized limitations of current therapies and the unmet need for effective treatments with demonstrated long-term tolerability, these data support the therapeutic utility of arbaclofen ER in patients with multiple sclerosis–related spasticity.

## Supplementary Material

fcad026_Supplementary_Data

## Abbreviations

EDSS =: Expanded Disability Status Scale
ER =: extended-release
GABA_B_ =: gamma-aminobutyric acid B
MAL =: most affected limb
PGIC =: Patient Global Impression of Change
SD =: standard deviation
TNmAS-MAL =: Total Numeric-transformed Modified Ashworth Scale in the most affected limb

## References

[1] Tullman MJ . Overview of the epidemiology, diagnosis, and disease progression associated with multiple sclerosis. Am J Manag Care. 2013;19(2 Suppl):S15–S20. 23544716

[2] Wallin MT , Culpepper WJ, Campbell JD, et al The prevalence of MS in the United States: A population-based estimate using health claims data. Neurology. 2019;92(10):e1029–e1040. 30770430 10.1212/WNL.0000000000007035 PMC6442006

[3] Hugos CL , Cameron MH. Assessment and measurement of spasticity in MS: State of the evidence. Curr Neurol Neurosci Rep. 2019;19(10):79. 31471769 10.1007/s11910-019-0991-2 PMC6948104

[4] Bethoux F , Marrie RA. A cross-sectional study of the impact of spasticity on daily activities in multiple sclerosis. Patient. 2016;9:537–546. 27154536 10.1007/s40271-016-0173-0

[5] Rizzo MA , Hadjimichael OC, Preiningerova J, Vollmer TL. Prevalence and treatment of spasticity reported by multiple sclerosis patients. Mult Scler. 2004;10(5):589–595. 15471378 10.1191/1352458504ms1085oa

[6] Amatya B , Khan F, La Mantia L, Demetrios M, Wade DT. Non-pharmacological interventions for spasticity in multiple sclerosis. Cochrane Database Syst Rev. 2013;2, CD009974 10.1002/14651858.CD009974.pub2. 10.1002/14651858.CD009974.pub2 PMC11813164 23450612

[7] Hughes C , Howard IM. Spasticity management in multiple sclerosis. Phys Med Rehabil Clin N Am. 2013;24(4):593–604. 24314678 10.1016/j.pmr.2013.07.003

[8] Henze T , Rieckmann P, Toyka KV, Multiple Sclerosis Therapy Consensus Group of the German Multiple Sclerosis Society. Symptomatic treatment of multiple sclerosis. Multiple Sclerosis Therapy Consensus Group (MSTCG) of the German Multiple Sclerosis Society. Eur Neurol. 2006;56(2):78–105. 16966832 10.1159/000095699

[9] Clemente CD . Neurophysiologic mechanisms and neuroanatomic substrates related to spasticity. Neurology. 1978; 28(9 Pt 2):40–45. 152417 10.1212/wnl.28.9_part_2.40

[10] Pozzilli C . Overview of MS spasticity. Eur Neurol. 2014;71(suppl 1):1–3. 24457845 10.1159/000357739

[11] Ertzgaard P , Campo C, Calabrese A. Efficacy and safety of oral baclofen in the management of spasticity: A rationale for intrathecal baclofen. J Rehabil Med. 2017;49(3):193–203. 28233010 10.2340/16501977-2211

[12] Sachais BA , Logue JN, Carey MS. Baclofen, a new antispastic drug: A controlled, multicenter trial in patients with multiple sclerosis. Arch Neurol. 1977;34(7):422–428. 327987 10.1001/archneur.1977.00500190056008

[13] Feldman RG , Kelly-Hayes M, Conomy JP, et al Baclofen for spasticity in multiple sclerosis: Double-blind crossover and three-year study. Neurology. 1978;28(11):1094–1098. 362234 10.1212/wnl.28.11.1094

[14] Sawa GM , Paty DW. The use of baclofen in treatment of spasticity in multiple sclerosis. Can J Neurol Sci. 1979;6:351–354. 385132 10.1017/s0317167100023994

[15] Dario A , Tomei G. A benefit-risk assessment of baclofen in severe spinal spasticity. Drug Saf. 2004;27:799–818. 15350152 10.2165/00002018-200427110-00004

[16] Olpe HR , Demieville H, Baltzer V, et al The biological activity of d- and l-baclofen (lioresal). Eur J Pharmacol. 1978;52(1):133–136. 214308 10.1016/0014-2999(78)90032-8

[17] Johnston GA , Hailstone MH, Freeman CG, et al Baclofen: Stereoselective inhibition of excitant amino acid release. J Pharm Pharmacol. 1980;32:230–231. 6103949 10.1111/j.2042-7158.1980.tb12902.x

[18] Terrence CF , Sax M, Fromm GH, Chang CH, Yoo CS. Effect of baclofen enantiomorphs on the spinal trigeminal nucleus and steric similarities of carbamazepine. Pharmacology. 1983;27(2):85–94. 6611651 10.1159/000137839

[19] Fromm CH , Terrence CF. Comparison of L-baclofen and racemic baclofen in trigeminal neuralgia. Neurology. 1987;37(11):1725–1728. 3313099 10.1212/wnl.37.11.1725

[20] Falch E , Hedegaard A, Nielsen L, et al Comparative stereostructure-activity studies on GABAA and GABAB receptor sites and GABA uptake using rat brain membrane preparations. J Neurochem. 1986;47(3):898–903. 3016189 10.1111/j.1471-4159.1986.tb00695.x

[21] Bowery NG , Hill DR, Hudson AL. (-) Baclofen decreases neurotransmitter release in the mammalian CNS by an action at a novel GABA receptor. Nature. 1980;283:92–94. 6243177 10.1038/283092a0

[22] Smith DF . Stereoselectivity of spinal neurotransmission: Effects of baclofen enantiomer on tail-flick reflex in rats. J Neural Transm. 1984;60(1):63–67. 6090584 10.1007/BF01254766

[23] Bowery NG . GABAB receptor pharmacology. Annu Rev Pharmacol Toxicol. 1993;33:109–147. 8388192 10.1146/annurev.pa.33.040193.000545

[24] Polman CH , Reingold SC, Banwell B, et al Diagnostic criteria for multiple sclerosis: 2010 revisions to the McDonald criteria. Ann Neurol. 2011;69(2):292–302. 21387374 10.1002/ana.22366 PMC3084507

[25] Haab F , Richard F, Amarenco G, et al Comprehensive evaluation of bladder and urethral dysfunction symptoms: Development and psychometric validation of the Urinary Symptom Profile (USP) Questionnaire. Urology. 2008;71(4):646–656. 18313122 10.1016/j.urology.2007.11.100

[26] Posner K , Brent D, Lucas C, et al Columbia-Suicide Severity Rating Scale (C-SSRS). Version 1/14/09. Available at https://depts.washington.edu/ebpa/sites/default/files/C-SSRS-LifetimeRecent-Clinical.pdf . Accessed August 21, 2020.

[27] Ashworth B . Preliminary trial of carisoprodol in multiple sclerosis. Practitioner. 1964;192:540–542. 14143329

[28] Bohannon RW , Smith MB. Interrater reliability of a modified Ashworth scale of muscle spasticity. Phys Ther. 1987;67(2):206–207. 3809245 10.1093/ptj/67.2.206

[29] Kurtzke J . Rating neurologic impairment in multiple sclerosis: An expanded disability status scale (EDSS). Neurology. 1983;33(11):1444–1452. 6685237 10.1212/wnl.33.11.1444

[30] Kantor D , deVries T, Dentiste A, et al Efficacy and safety of arbaclofen extended-release tablets in the treatment of multiple sclerosis spasticity (Study OS440–3002). Poster presented at: 33rd Annual Meeting of the Consortium of Multiple Sclerosis Centers; May 28—June 1, 2019; Seattle, Washington. Poster SXM07.

[31] Hunter SF , deVries T, Dentiste A, et al One-year study to evaluate the long-term safety of arbaclofen extended-release in multiple sclerosis-related spasticity (Study OS440–3003). Poster presented at: 33rd Annual Meeting of the Consortium of Multiple Sclerosis Centers; May 28—June 1, 2019; Seattle, Washington. Poster SXM08.

[32] United Kingdom Tizanidine Trial Group . A double-blind, placebo-controlled trial of tizanidine in the treatment of spasticity caused by multiple sclerosis. Neurology. 1994;44(11 Suppl 9):S70–S78. 7970014

