# Supplementary material for: Arbaclofen extended-release tablets for spasticity in multiple sclerosis: open-label extension study
Source: Brain Commun. 2023 Feb 7;5(1):fcad026. doi: 10.1093/braincomms/fcad026 (PMC9968651; doi:10.1093/braincomms/fcad026)
Supplement: fcad026_Supplementary_Data [file fcad026_supplementary_data.docx]

Arbaclofen Extended-Release Tablets for Spasticity in Multiple Sclerosis: Open-label Extension Study

Darin T. Okuda, MD; Daniel Kantor, MD; Mark Jaros, PhD;
Tina deVries, PhD; Samuel Hunter, MD, PhD

Supplementary Materials

Supplementary Methods Enrollment criteria

Supplementary Figure 1 Enrollment and disposition of patients in the open-label extension study

Supplementary Table 1 Patient disposition and reasons for discontinuation in the open-label extension study

Supplementary Table 2 Reasons for discontinuation in the open-label extension study by prior arbaclofen exposure

Supplementary Table 3 USP Questionnaire scores—open-label extension study

Supplementary Table 4 Longitudinal analysis of TNmAS scores in patients who completed the randomized controlled trial and enrolled in the open-label extension study

Reference

Supplementary Methods. Enrollment criteria

Inclusion criteria

Male and female patients will be considered eligible for participation in the study if all the following inclusion criteria are satisfied at Visit 1 (baseline) for *de novo* patients and at the last visit of the randomized controlled trial for rollover patients:

1. Patients 18 to 65 years of age, inclusive.

2. An established diagnosis of multiple sclerosis per McDonald Criteria^1^ (either relapsing–remitting or secondary–progressive course) with a documented history of spasticity for at least 6 months prior to baseline.

3. Has participated in Study OS440-3004 or is a new U.S. patient (a *de novo* patient) who fulfills the inclusion/exclusion criteria.

a. *De novo* patients being considered for enrollment must have spasticity due to multiple sclerosis as shown by a TNmAS score ≥2 in the most affected limb.

4. Is willing to continue on open-label treatment with arbaclofen extended-release (ER) as described in the protocol.

5. If receiving disease-modifying medications (eg, interferons approved for multiple sclerosis, glatiramer acetate, natalizumab, fingolimod, or mitoxantrone), there must be no change in dose for at least 3 months prior to baseline, and the patient must be willing to maintain this treatment dose for the duration of the study. If receiving Ampyra^®^ (dalfampridine, fampridine, 4-amino pyridine), the patient must be at a stable dose for at least 3 months prior to baseline.

6. Stable regimen for at least 1 month prior to baseline for all medications and non-pharmacological therapies that are intended to alleviate spasticity.

a. *De novo* patients being considered for enrollment and taking medications indicated for the treatment of spasticity (ie, baclofen, benzodiazepines, cannabinoids, carisoprodol, dantrolene, tizanidine, cyclobenzaprine, any neuroleptic, ropinirole, tolperisone, and clonidine) must wash out from these medications for at most 21 days by baseline to be eligible for study treatment. *De novo* patients found not to meet this criterion will be withdrawn from the study and will be considered screen failures.

7. Absence of infections, peripheral vascular disease, painful contractures, advanced arthritis, or other conditions that hinder evaluation of joint movement.

8. Creatinine clearance as calculated by the GFR using the MDRD formula of >50 mL/minute.

9. Use of a medically highly effective form of birth control during the study and for 3 months thereafter for women of child-bearing potential.

10. Willing to sign the informed consent form.

Exclusion criteria

Patients who meet any of the following criteria will not qualify for the study:

1. Any concomitant disease or disorder that has symptoms of spasticity or that may influence the patient’s level of spasticity.

2. Inability to rate their level of spasticity or distinguish it from other multiple sclerosis symptoms.

3. Use of high dose oral or intravenous methylprednisolone, or equivalent, within 3 months before baseline.

4. History of allergy to baclofen or any inactive components of the test formulation.

5. Concomitant use of medications that would potentially interfere with the actions of the study medication or outcome variables.

6. Pregnancy, lactation, or planned pregnancy during the course of the study and for 3 months after the final study visit.

7. Recent history (within past 12 months) of any unstable psychiatric disease (or yes response to questions 1 or 2 on the C-SSRS at baseline), or current signs and symptoms of significant medical disorders such as severe, progressive, or uncontrolled pulmonary, cardiac, gastrointestinal, hepatic, renal, genitourinary, hematological, endocrine, immunologic, or neurological disease.

8. History of epilepsy.

9. Current significant cognitive deficit, severe or untreated anxiety, severe or untreated depression.

10. Patients with abnormal micturition that requires indwelling or intermittent catheterization or with lower urinary tract symptoms that result in a score >26 in the baseline USP^©^ Questionnaire. Patients who are proficient in self-catheterization may be included in the study at the investigator’s discretion.

11. Current malignancy or history of malignancy that has not been in remission for more than 5 years, except effectively-treated basal cell skin carcinoma.

12. Clinically significant abnormal laboratory values, in the opinion of the investigator at screening (at Visit 6 for rollover patients).

13. Any other significant disease, disorder, or significant laboratory finding, including clinically significant abnormal laboratory values or ongoing serious adverse events at Visit 6 (Final Visit) of Study OS440-3004, which, in the opinion of the investigator, puts the patient at risk because of participation, influences the result of the study, or affects the patient’s ability to participate.

14. Planned elective surgery or other procedures requiring general anesthesia during the course of the study.

15. History of any illicit substance abuse (eg, alcohol, marijuana, cocaine) or prescription for long-acting opioids within the past 12 months (tramadol use will be allowed).

16. Participation in another clinical research study (with the exception of Study OS440-3004) within 1 month of baseline.

Supplementary Figure 1. Enrollment and disposition of patients in the open-label extension study

Assessed for eligibility (n=328)

*De novo* patients (n=11)

Rollover from randomized trial (n=317)

Excluded (n=5)

Did not meet eligibility criteria (n=5)

*De novo* (n=6)
 ≤40 mg/day (n=2)
 80 mg/day (n=4)

From placebo (n=136)
 40 mg/day (n=20)
 60 mg/day (n=21)
 80 mg/day (n=95)

From 40 mg/day (n=107)
 40 mg/day (n=17)
 60 mg/day (n=13)
 80 mg/day (n=77)

From 80 mg/day (n=74)
 40 mg/day (n=6)
 60 mg/day (n=5)
 80 mg/day (n=63)

^a^Includes both treatment-emergent and non–treatment-emergent adverse events.
Abbreviations: MS, multiple sclerosis.

Discontinued (n=27)

Patient request (n=13)

Adverse event (n=6)^a^

MS relapse (n=5)

Other (n=3) 

Discontinued (n=64)

Patient request (n=29)

Adverse event (n=29)^a^

MS relapse (n=5)

Other (n=1) 

Discontinued (n=2)

Adverse event (n=2)^a^

Discontinued (n=12)

Patient request (n=8)

Adverse event (n=3)^a^

Other (n=1) 

Completed open label study (n=218)

Treatment-naïve (n=142)

Treatment-experienced (n=181)

Enrolled in open-label study (n=323)

Supplementary Table 1. Patient disposition and reasons for discontinuation in the open-label extension study

| Arbaclofen ER maintenance dose | Arbaclofen ER  <40 mg/day  (n=1) | Arbaclofen ER  40 mg/day  (n=44) | Arbaclofen ER  60 mg/day  (n=39) | Arbaclofen ER  80 mg/day  (n=239) | Total  population  (N=323) |
| --- | --- | --- | --- | --- | --- |
| Completed study treatment | 0 | 26 (59.1) | 20 (51.3) | 172 (72.0) | 218 (67.5) |
| Discontinued treatment early | 1 (100) | 18 (40.9) | 19 (48.7) | 67 (28.0) | 105 (32.5) |
| Reasons for discontinuation | | | | | |
| Adverse events  Relapse  TEAE  Non–TEAE | 1 (100)  0  1 (100)  0 | 9 (20.5)  2 (4.5)  7 (15.9)  0 | 16 (41.0)  3 (7.7)  13 (30.8)^a^  0^a^ | 24 (10.0)  5 (2.1)  17 (7.1)  2 (0.8) | 50 (15.5)^a^  10 (3.0)^b^  38 (11.8)^a^  2 (0.6) |
| Patient request | 0 | 9 (20.5) | 3 (7.7) | 38 (15.9) | 50 (15.2) |
| Medical condition | 0 | 0 | 0 | 1 (0.4) | 1 (0.3) |
| Investigator decision | 0 | 0 | 0 | 1 (0.4) | 1 (0.3) |
| Other | 0 | 0 | 0 | 3 (1.3) | 3 (0.9) |

^a^One patient who discontinued the study with a TEAE and a non-TEAE counted in discontinued-due-to-TEAE row

^b^For the purposes of the study protocol, patients who experienced an MS-relapse were considered to have an adverse event.

ER = extended-release; TEAE = treatment-emergent adverse event.

Supplementary Table 2. Reasons for discontinuation in the open-label extension study by prior arbaclofen exposure

| Arbaclofen exposure status | Treatment-experienced^a^  (n=181) | Treatment-naïve^b^  (n=142) |
| --- | --- | --- |
| Completed study treatment | 142 (78.5) | 76 (53.5) |
| Discontinued treatment early | 39 (21.5) | 66 (46.5) |
| Reasons for discontinuation |  | |
| Adverse events^c^  Relapse  All other | 14 (7.7)  5 (2.8)  9 (5.0) | 36 (25.4)  5 (3.5)  31 (21.8) |
| Patient request | 21 (11.6) | 29 (20.4) |
| Medical condition | 1 (0.6) | 0 |
| Investigator decision | 1 (0.6) | 0 |
| Other | 2 (1.1) | 1 (0.7) |

^a^Includes rollover patients who received arbaclofen ER at any dose in the randomized controlled trial.

^b^Includes rollover patients who received placebo in the randomized controlled trial plus *de novo* patients.

^c^ Includes both treatment-emergent adverse events and non–treatment-emergent adverse events.

ER = extended-release.

Supplementary Table 3. USP Questionnaire scores—open-label extension study

| Parameter, mean (95% CI) | Arbaclofen ER  40 mg/day  (n=44) | Arbaclofen ER  60 mg/day  (n=39) | Arbaclofen ER  80 mg/day  (n=239) | Total  population  (N=323)^a^ |
| --- | --- | --- | --- | --- |
| Stress urinary incontinence score | | | | |
| Baseline | 1.4 (0.7, 2.0) | 0.9 (0.3, 1.5) | 1.3 (1.0, 1.6) | 1.3 (1.0, 1.5) |
| Week 28 | 1.8 (0.9, 2.6) | 0.4 (–0.1, 1.0) | 1.3 (1.0, 1.6) | 1.3 (1.0, 1.5) |
| Week 28 change from baseline | 0.3 (–0.2, 0.8) | 0.2 (–0.2, 0.6) | 0.0 (–0.2, 0.2) | 0.1 (–0.1, 0.2) |
| Week 60/final visit | 2.4 (1.1, 3.6) | 0.6 (–0.2, 1.4) | 1.2 (0.9, 1.5) | 1.3 (1.0, 1.6) |
| Week 60/final visit change from baseline | 0.6 (–0.3, 1.6) | 0.5 (–0.4, 1.3) | –0.1 (–0.3, 0.1) | 0.0 (–0.2, 0.3) |
| Overactive bladder score | | | | |
| Baseline | 5.2 (3.9, 6.5) | 4.4 (3.2, 5.6) | 5.2 (4.7, 5.7) | 5.1 (4.6, 5.5) |
| Week 28 | 4.6 (3.2, 5.9) | 4.0 (2.4, 5.7) | 5.2 (4.7, 5.7) | 5.0 (4.5, 5.5) |
| Week 28 change from baseline | –0.2 (–0.9, 0.4) | 0.7 (–0.1, 1.6) | 0.0 (–0.4, 0.4) | 0.0 (–0.3, 0.3) |
| Week 60/final visit | 5.5 (3.4, 7.5) | 4.0 (2.2, 5.8) | 5.0 (4.4, 5.6) | 5.0 (4.4, 5.5) |
| Week 60/final visit change from baseline | –0.1 (–1.3, 1.2) | 0.8 (–0.4, 2.0) | –0.3 (–0.7, 0.1) | –0.2 (–0.5, 0.2) |
| Low stream score | | | | |
| Baseline | 1.5 (1.0, 1.9) | 1.2 (0.8, 1.6) | 1.5 (1.3, 1.7) | 1.5 (1.3, 1.6) |
| Week 28 | 1.3 (0.8, 1.8) | 1.1 (0.5, 1.7) | 1.5 (1.3, 1.8) | 1.5 (1.3, 1.7) |
| Week 28 change from baseline | –0.1 (–0.3, 0.1) | –0.3 (–0.7, 0.0) | 0.0 (–0.2, 0.2) | 0.0 (–0.2, 0.1) |
| Week 60/final visit | 1.3 (0.7, 1.9) | 1.6 (0.8, 2.3) | 1.6 (1.4, 1.8) | 1.6 (1.4, 1.8) |
| Week 60/final visit change from baseline | –0.2 (–0.5, 0.1) | 0.1 (–0.4, 0.5) | 0.0 (–0.2, 0.2) | 0.0 (–0.2, 0.2) |

^a^Includes 1 patient who received <40 mg and discontinued treatment due to nausea categorized as a moderate adverse event.

CI = confidence interval; ER = extended-release; USP = Urinary Symptom Profile.

Supplementary Table 4. Longitudinal analysis of TNmAS Scores in patients who completed the randomized controlled trial and enrolled in the open-label extension study

| Outcome | Arbaclofen ER  40 mg/day  (n=43) | Arbaclofen ER  60 mg/day  (n=39) | Arbaclofen ER  80 mg/day  (n=235) | Total  population  (n=317) |
| --- | --- | --- | --- | --- |
| TNmAS–MAL score, mean (SD) | | | | |
| Baseline, RCT | 6.4 (3.42) | 7.3 (3.04) | 8.0 (2.99) | 7.7 (3.10) |
| OLE study Week 28 change from RCT baseline | –1.3 (2.32) | –1.7 (2.07) | –2.2 (2.12) | –2.0 (2.16) |
| OLE study Week 60 change from RCT baseline^a^ | –1.1 (1.69) | –1.5 (3.05) | –1.6 (2.07) | –1.5 (2.13) |
| TNmAS–TL score, mean (SD) | | | | |
| Baseline, RCT | 12.9 (8.69) | 14.5 (8.71) | 16.7 (7.93) | 15.9 (8.23) |
| OLE study Week 28 change from RCT baseline | –3.0 (5.22) | –3.5 (5.08) | –4.4 (5.16) | –4.1 (5.17) |
| OLE study Week 60 change from RCT baseline^a^ | –1.5 (4.00) | –2.4 (6.40) | –2.8 (5.16) | –2.6 (5.15) |

^a^Assessment performed 4 weeks after completion of taper from study drug.

ER = extended-release; OLE = open-label extension; RCT = randomized controlled trial; SD = standard deviation; TNmAS–MAL = Total Numeric-modified Ashworth Scale–Most Affected Limb; TNmAS–TL = Total Numeric-modified Ashworth Scale–Total Limbs.

Reference

1. Polman CH, Reingold SC, Banwell B, et al. Diagnostic criteria for multiple sclerosis: 2010 revisions to the McDonald criteria. *Ann Neurol*. 2011;69(2):292–302.
